# Changes in Corticomotor Excitability and Intracortical Inhibition of the Primary Motor Cortex Forearm Area Induced by Anodal tDCS

**DOI:** 10.1371/journal.pone.0101496

**Published:** 2014-07-07

**Authors:** Xue Zhang, Daniel G. Woolley, Stephan P. Swinnen, Hilde Feys, Raf Meesen, Nicole Wenderoth

**Affiliations:** 1 Motor Control Laboratory, Movement Control and Neuroplasticity Research Group, Department of Kinesiology, KU Leuven, Leuven, Belgium; 2 Research Group for Neuromotor Rehabilitation, Department of Rehabilitation Sciences, KU Leuven, Leuven, Belgium; 3 REVAL Rehabilitation Research Centre, Biomedical Research Institute, Hasselt University, Hasselt, Belgium; 4 Neural Control of Movement Laboratory, Department of Health Sciences and Technology, ETH Zurich, Zurich, Switzerland; University of Toronto, Canada

## Abstract

**Objective:**

Previous studies have investigated how tDCS over the primary motor cortex modulates excitability in the intrinsic hand muscles. Here, we tested if tDCS changes corticomotor excitability and/or cortical inhibition when measured in the extensor carpi radialis (ECR) and if these aftereffects can be successfully assessed during controlled muscle contraction.

**Methods:**

We implemented a double blind cross-over design in which participants (n = 16) completed two sessions where the aftereffects of 20 min of 1 mA (0.04 mA/cm^2^) anodal vs sham tDCS were tested in a resting muscle, and two more sessions where the aftereffects of anodal vs sham tDCS were tested in an active muscle.

**Results:**

Anodal tDCS increased corticomotor excitability in ECR when aftereffects were measured with a low-level controlled muscle contraction. Furthermore, anodal tDCS decreased short interval intracortical inhibition but only when measured at rest and after non-responders (n = 2) were removed. We found no changes in the cortical silent period.

**Conclusion:**

These findings suggest that targeting more proximal muscles in the upper limb with anodal tDCS is achievable and corticomotor excitability can be assessed in the presence of a low-level controlled contraction of the target muscle.

## Introduction

Transcranial direct current stimulation (tDCS) is the noninvasive application of a weak electrical current to brain tissue. The aftereffects of tDCS can last in the order of minutes to hours, depending on the length and intensity of stimulation [1]. Considerable effort has been directed towards highlighting the positive effect of tDCS on behavior, with studies demonstrating improved motor learning in healthy controls [2]–[5] and enhanced neurorehabilitation outcomes in patients recovering from stroke [6]–[8].

Although the underlying physiological mechanisms through which tDCS exerts its effect are not yet fully understood, its application to primary motor cortex appears to alter the polarization of resting membrane potentials, reflected by measurable short term changes in corticomotor excitability[9], [10]. It has been postulated that the long-term effects of tDCS on motor behaviour might occur through the modulation of synaptic plasticity in the motor cortex [5]. Evidence suggests that these long-term effects are dependent on the modulation of NMDA receptor-dependent long-term potentiation and GABAergic inhibition [10]–[13].

The application of anodal tDCS to the primary motor cortex significantly increases corticomotor excitability and decreases intracortical inhibition in both healthy [1], [9], [11], [14]–[19] and patient populations [6], [7], [20]. These findings in particular suggest that tDCS might prove useful as a post stroke rehabilitation tool. Most existing research has focused on examining how tDCS modulates corticomotor excitability and intracortical inhibition in the hand area of primary motor cortex [11], [15], [21]. However, the restoration of wrist function and wrist extension in particular, is equally critical during stroke rehabilitation [22]. A second consideration regarding how well previous work generalizes to clinical settings is highlighted when considering how tDCS aftereffects are typically measured. Single pulse transcranial magnetic stimulation (TMS) is used to induce motor evoked potentials (MEPs) before and after stimulation in order to assess corticomotor excitability [1], [9], and a short-interval paired pulse TMS protocol is used to measure intracortical inhibition [6], [11], [15], [20], [23], with both measurements often performed with the target muscle in a resting state [1], [9], [11], [14], [15], [18], [21], [24]. However, in moderately to severely impaired stroke patients, TMS induced MEPs are often absent in the paretic limb when muscles are relaxed making it difficult to asses tDCS aftereffects in this patient group [25]. Here we tested the influence of 20 min anodal tDCS (1 mA) over the primary motor cortex on the corticomotor excitability of the wrist extensor muscle in healthy adults. TDCS aftereffects were tested either at rest or while subjects maintained a low level isometric wrist extension. Based on previous work that mainly demonstrated tDCS effects in finger muscles [6]–[8] we hypothesize that anodal tDCS applied to the primary motor cortex induces measurable aftereffects in the corticomotor excitability of wrist extensor muscles, and that these aftereffects are also present when measured during light contraction of the target muscle.

## Methods

### 1. Ethics statement

All experimental procedures were approved by the local Ethics Committee for Biomedical Research at the KU Leuven (ethics approval numer: S52763) and conformed to the Declaration of Helsinki (1964).

### 2. Participants

Sixteen healthy male adults (mean age and standard deviation: 21.5±1.31 yr, range: 20–24 yr) participated in the experiment after providing written informed consent. All participants were right-handed [26] (mean Laterality index and standard deviation 80±27%, range: 20–100%). None of the participants reported contraindications to TMS or tDCS, and all were free of medication, had no history of neurological or psychiatric disease and were naïve to the purpose of the experiment. We did not include females because our protocol involved repeated measurements that were separated by at least a week, which might be influenced by the hormonal cycle [27].

### 3. Study design

**Figure 1:**
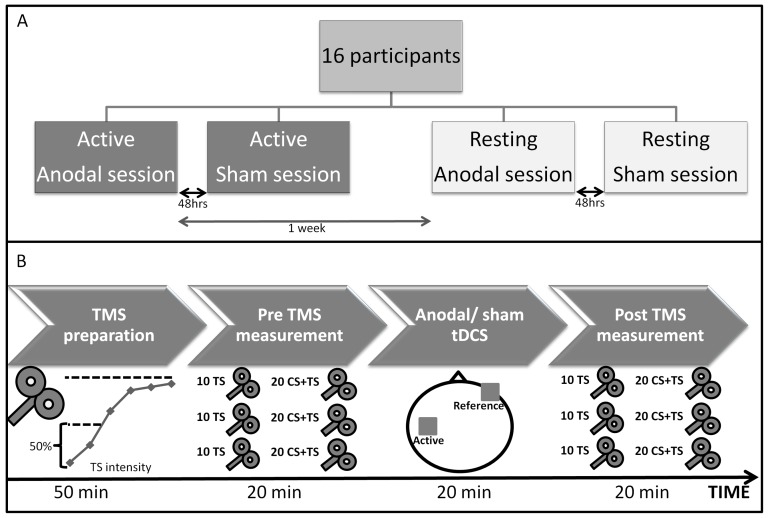
Study protocol. **A**: Each participant was tested in 4 separate sessions: 2 active sessions (anodal and sham) and 2 resting sessions (anodal and sham). There was at least 1 week break between 2 consecutive anodal sessions and 48 hrs break between other sessions. The order of testing was counterbalanced. **B**: Test session procedure. An IO-curve was measured to determine the test stimulation (TS) intensity defined as 50% of the maximum MEP amplitude. Pre and post TMS measurement consisted of 3 blocks of 30 TMS pulses. Each block consisted of 10 single pulse (measuring corticomotor excitability) and 20 double pulse (measuring SICI) TMS measurements. Anodal/sham tDCS was applied for 20 min over the left primary motor cortex with 1 mA intensity.

We implemented a double blind cross-over design, where each participant completed 4 separate test sessions: 2 active sessions, which probed the effect of anodal versus sham tDCS on corticomotor excitability and intracortical inhibition during a controlled muscle contraction, and 2 resting sessions which probed the effect of anodal versus sham tDCS on corticomotor excitability and intracortical inhibition during rest. The order of motor state (active and rest) and stimulation type (anodal and sham) were counterbalanced across participants. There was at least 48 hrs between anodal and sham sessions and 1 week between consecutive anodal sessions (Fig. 1A). In each test session, participants followed the same procedure: 1) TMS preparation, 2) pre TMS measurement (PRE-TMS), 3) anodal/sham tDCS stimulation and 4) post TMS measurement (POST-TMS, Fig. 1B). Please note that tDCS was always applied while the subject was at rest, while the assessment of tDCS aftereffect was either performed in the relaxed or the pre-contracted state.

#### 3.1 General setup

Participants were comfortably seated with their right forearm abducted and fixed to a custom made device that only allowed movement about the wrist joint. Wrist extension force was measured by a force sensor (Load cell model 1042, TEDEA Huntleigh, USA). Visual feedback of wrist extension force amplitude was provided during the active sessions. MEPs were recorded from the right ECR with surface electromyography (EMG; Mespec 8000, Mega Electronic, UK). Two disposable Ag-AgCl surface electrodes (Blue Sensor P-00-S, Ambu, Denmark) were placed in a belly-tendon montage on the right ECR, with the reference electrode placed on the lateral epicondyle. In the first session of each condition (i.e. active versus rest), the EMG electrodes positions were marked with a semi permanent marker to ensure identical positioning of EMG electrodes across the anodal and sham sessions. EMG data were sampled at 5000 Hz (CED Power 1401, Cambridge Electronic Design, UK), amplified, band pass filtered (5–1000 Hz), and stored on a PC for off-line analysis.

In the active sessions, each subject's isometric wrist extension maximal voluntary contraction (MVC) was recorded at the beginning of the session. MVC was taken as the highest value of three maximal isometric contractions.

#### 3.2 TMS preparation

TMS was performed with a figure-of-eight coil (loop diameter 70 mm) connected to two Magstim Bi-Stim^2^ stimulators (Magstim, UK). The coil was positioned over the hotspot of ECR (i.e. the location with the largest and most consistent MEPs) with the optimal orientation (i.e. the coil was positioned over the left hemisphere, tangentially to the scalp with the handle pointing backward and laterally at 45° away from the mid-sagittal line) for evoking a descending volley in the corticospinal tract. The same coil orientation was used for all subjects. In the first session of each condition (i.e. active versus rest), the hotspot was determined and marked with a semi permanent marker. In the following sessions, we confirmed that the previously defined hotspot was the point where the largest and most consistent MEPs were evoked prior to the main measurements. Accordingly, the hotspot location was highly consistent across sessions. The average location in each session with respect to the lateral and anterior distance from the vertex was rest-anodal 5.2±0.1 cm, 0.5±0.2 cm; rest-sham: 5.2±0.1 cm, 0.5±0.2 cm; active-anodal: 5.3± 0.2 cm, 0.3±0.2 cm; active-sham: 5.2± 0.2 cm, 0.6± 0.2 cm.

Rest motor threshold (RMT), defined as the lowest stimulus intensity eliciting MEPs >50µV in at least five out of 10 consecutive trials, was determined to the nearest 1% of maximum stimulator output. Active motor threshold (AMT) was determined as the lowest stimulus intensity eliciting MEPs >200 µV in at least five out of 10 consecutive trials while participants contracted their wrist extensor with an isometric force of 10% MVC (i.e. MEP size had to exceed the background EMG and was usually followed by a silent period).

TMS stimulation intensities were determined in a similar manner to that previously described by Byblow et al [28]. In brief, an input-output curve (IO-curve) was measured prior to the main experiment in each test session. In the resting sessions, the IO-curve was determined using stimulation intensities ranging from 90%–190% of RMT in steps of 20%. Five MEPs were collected per intensity (30 in total) in a randomized order with an inter-trial interval varying randomly between 7 to 9 s. The total procedure lasted approximately 4 min. In the active sessions, the IO-curve was measured at intensities ranging from 90%–210% of AMT in steps of 20%. Five MEPs were collected per intensity (35 in total) in a randomized order and with an inter-trial interval between 7 and 9 s (corresponding to approximately 5 min in total). Based on the IO-curve, the suprathreshold test stimulus (TS) intensity was set to evoke MEPs with an amplitude corresponding to 50% of maximum.

Short interval intracortical inhibition (SICI) was measured using paired pulse TMS such that the TS was preceded by a subthreshold conditioning stimulus (CS) with the interstimulus interval (ISI) being set to 3 ms [11], [15], [16], [23]. MEP amplitude is diminished when the TS is preceded by the CS (CS+TS) indicating the influence of inhibitory circuits. However, it has been shown that this MEP reduction depends on the CS intensity and that inhibitory activity can be estimated more reliably when several CS intensities are used, i.e. a SICI curve is measured [29], [30]. Here, three CS intensities were used: For each subject a CS intensity of 80% of motor threshold (MT; i.e. 80% AMT for active condition, 80% RMT for resting condition) was used as a starting reference. We then determined the stimulator output (to the nearest 1%) that maximally reduced the MEP amplitude (CS_max_). In addition to CS_max_, the SICI curve consisted of measurements at CS_max_±10% of MSO (CS_max-10%_, CS_max+10%_).

#### 3.3 Pre- and post- TMS measurement

Identical TMS measurements were executed prior to and immediately after the tDCS intervention: Three blocks of 10 unconditioned pulses (TS only, single pulse) and 20 conditioned pulses (SICI, paired pulse) were delivered. The order of CS intensity was varied randomly between blocks. Breaks of 2 min were included after each block.

**Figure 2:**
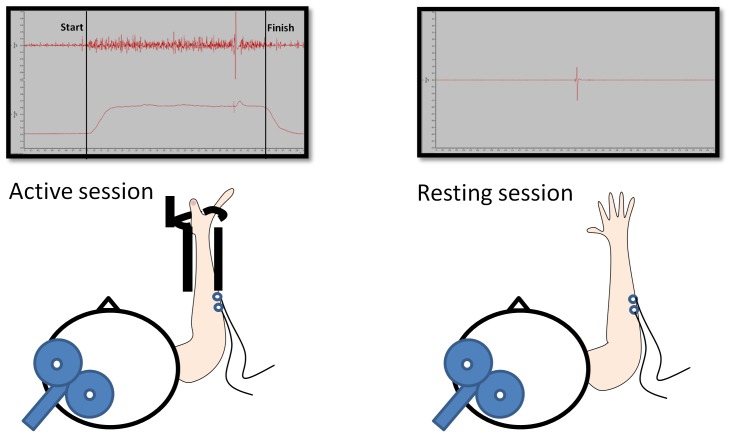
Testing setup. In the active sessions (left), subjects contracted their right ECR at 10% of maximum voluntary contraction during TMS measurement. In the resting sessions (right) subjects were passively resting their right arm on the table. Exemplary EMG activity is shown in the upper panels.

In the resting sessions participants were asked to relax their forearm and hand muscles, which were closely monitored via background EMG (bgEMG). In the active sessions participants contracted their wrist extensors for 3 s at 10% of MVC during each TMS measurement, with TMS given 2.5 s after each contraction (Fig. 2). Production of the required extension force was monitored by the experimenter. During all TMS measurements, the experimenters were blinded as to whether subjects received anodal or sham tDCS. In each session one experimenter was responsible for holding the TMS coil and communicating with the subject, one was responsible for controlling the data collection computer, and another observed. Programming of the tDCS stimulator for a given participant was performed by the experimenter who was not directly involved in testing that participant (the observer).

#### 3.4 tDCS intervention

TDCS stimulation (HDCstim-DC stimulator, Newronika, Italy) was delivered at 1 mA via two electrodes with a 5 cm×5 cm surface area resulting in a current density of 0.04 mA/cm^2^. The center of the anodal electrode was placed over the hotspot of the left ECR region of primary motor cortex (identified by TMS), while the reference electrode was fixed to the contralateral supraorbital ridge [9]. Electrodes were covered with conductive gel and then placed in saline soaked sponges. Pilot work indicated that this procedure was optimal for ensuring that low impedance between the electrodes and the scalp was maintained over the course of the experiment. In the anodal session, the current ramped up over 10 s and was maintained at 1 mA for 20 min. In the sham session, the current ramped up over 10 s and was maintained at 1 mA for 12 s, and then quickly faded. Participants were not able to distinguish between anodal and sham stimulation using this procedure.

At the end of each session, participants filled in a questionnaire to determine the level of physical activity before the test, the number of sleep hours and sleep quality during the previous night, and a discomfort score for the tDCS stimulation.

### 4. Data analyses

For all TMS measurements, MEP size was determined by peak-to-peak amplitude. MEPs were considered outliers and excluded from the analysis if they were greater than Q3+1.5× (Q3-Q1) or less than Q1-1.5× (Q3-Q1), where Q1 and Q3 are equal to the first and third quartiles, respectively. Single pulse MEPs were pooled separately for PRE-TMS and POST-TMS (30 MEPs for PRE-TMS and 30 MEPs for POST-TMS). BgEMG was quantified by the root mean square error of the EMG signal in an interval between 10 and 110 ms before TMS stimulation. For each participant, the mean and standard deviation (SD) of the bgEMG score was calculated separately for each session. In the resting sessions, trials with a bgEMG score greater than the mean + 2.5 SDs were removed from the analysis. In the active sessions, trials with a bgEMG score greater than the mean + 2.5 SDs and/or smaller than the mean −2.5 SDs were removed from the analysis. According to these criteria 95±3% of the trials were included in the analysis. All statistics were calculated with Statistica 11 (StatSoft, USA). The level of significance was set to α = 0.05.

#### 4.1 Corticomotor excitability

Changes in corticomotor excitability resulting from the tDCS intervention were quantified by calculating the average PRE-TMS and POST-TMS MEP sizes, and then calculating the POST-TMS/PRE-TMS MEP ratio, such that values greater than 1 indicate an increase in corticomotor excitability and values less than1 indicate a decrease. This allowed us to directly compare the effect of anodal tDCS between the active and resting sessions with a repeated measures analysis of variance (rm-ANOVA) including within subject factors MOTOR STATE (active, resting) and STIMULATION (anodal, sham). We also estimated the variability of the MEPs by determining the coefficient of variation (CV), which was analyzed with the same rm-ANOVA model.

#### 4.2 Short interval intracortical inhibition

Paired pulse TMS induced reductions in TS MEP amplitudes reflecting SICI, which were expressed as a percentage of the unconditioned MEP amplitude (values closer to 100% represent weaker intracortical inhibition). Sixty double pulse MEPs were collected separately for PRE-TMS and POST-TMS (20 double pulse MEPs for each CS intensity in PRE-/POST- TMS). SICI data were analyzed with an rm-ANOVA model including within subject factors MOTOR STATE (active, resting), STIMULATION (anodal, sham), TIME (pre, post) and CS intensity (CS_max-10%_, CS_max_, CS_max+10%_). However, since we expected a significant overall reduction in strength of SICI in the active state when compared to rest [31], we also calculated two separate rm-ANOVA models for each motor state with the factors STIMULATION, TIME and CS intensity.

#### 4.3 Background EMG

BgEMG scores were averaged separately for PRE-TMS and POST-TMS measurements and analyzed with a rm-ANOVA including within subject factors MOTOR STATE (active, resting), STIMULATION (anodal, sham) and TIME (pre, post).

#### 4.4 Cortical silent period and wrist extension force

In the active sessions, cortical silent period (cSP) and wrist extension force data were also analyzed following previously established procedures [32]–[34]. The cSP onset was defined as the time point when the TMS induced changes in EMG returned to baseline. The cSP offset was defined as the time point when voluntary EMG activity recovered and reached the same level as prestimulus bgEMG (referred to as the target range). The prestimulus bgEMG was collected between 5 and 105 ms before TMS stimulation. The target range of the bgEMG was determined as the mean ± the root mean square error of the prestimulus bgEMG. The cSP data and wrist extension force were averaged for PRE-TMS and POST-TMS measurements and analyzed with a rm-ANOVA including within subject factors STIMULATION (anodal, sham) and TIME (pre, post).

## Results

All participants completed all sessions of the study and none reported any adverse reaction to tDCS or TMS. There were no significant differences between stimulation intensities between anodal and sham test sessions in either active or resting motor states (Table 1).

**Table 1:** Stimulation intensities relative to the maximum stimulator output (MSO) and motor threshold (MT).

	CS_max_ intensity (% MSO)	TS intensity (% MSO)	CS_max_ intensity (% MT)	TS intensity (% MT)
**Rest-anodal**	32.00±1.25	50.63±2.21	85.59±2.02	135.01±2.82
**Rest-sham**	32.50±1.14	51.68±2.03	86.17±1.76	136.54±2.02
**Active-anodal**	33.00±1.05	48.88±1.88	88.99±1.51	131.43±2.35
**Active-sham**	32.81±1.11	49.19±1.99	88.51±1.97	132.38±3.02

Values are means ± SEM.

### 1. Corticomotor excitability

PRE-TMS represents a measure of baseline corticomotor excitability in each test session. The baseline MEP amplitudes for the active motor sessions were 2.11 mV±0.21 (mean ± SEM) and 2.42 mV±0.30 for anodal and sham respectively (paired *t*-test, anodal versus sham *t*_(15)_ = −1.67, p =  0.11). In the resting motor sessions, the baseline MEP amplitudes were 1.03 mV±0.15 and 0.97 mV±0.09 for anodal and sham respectively (paired *t*-test, anodal versus sham *t*_(15)_ = 0.58, p =  0.57).

**Figure 3:**
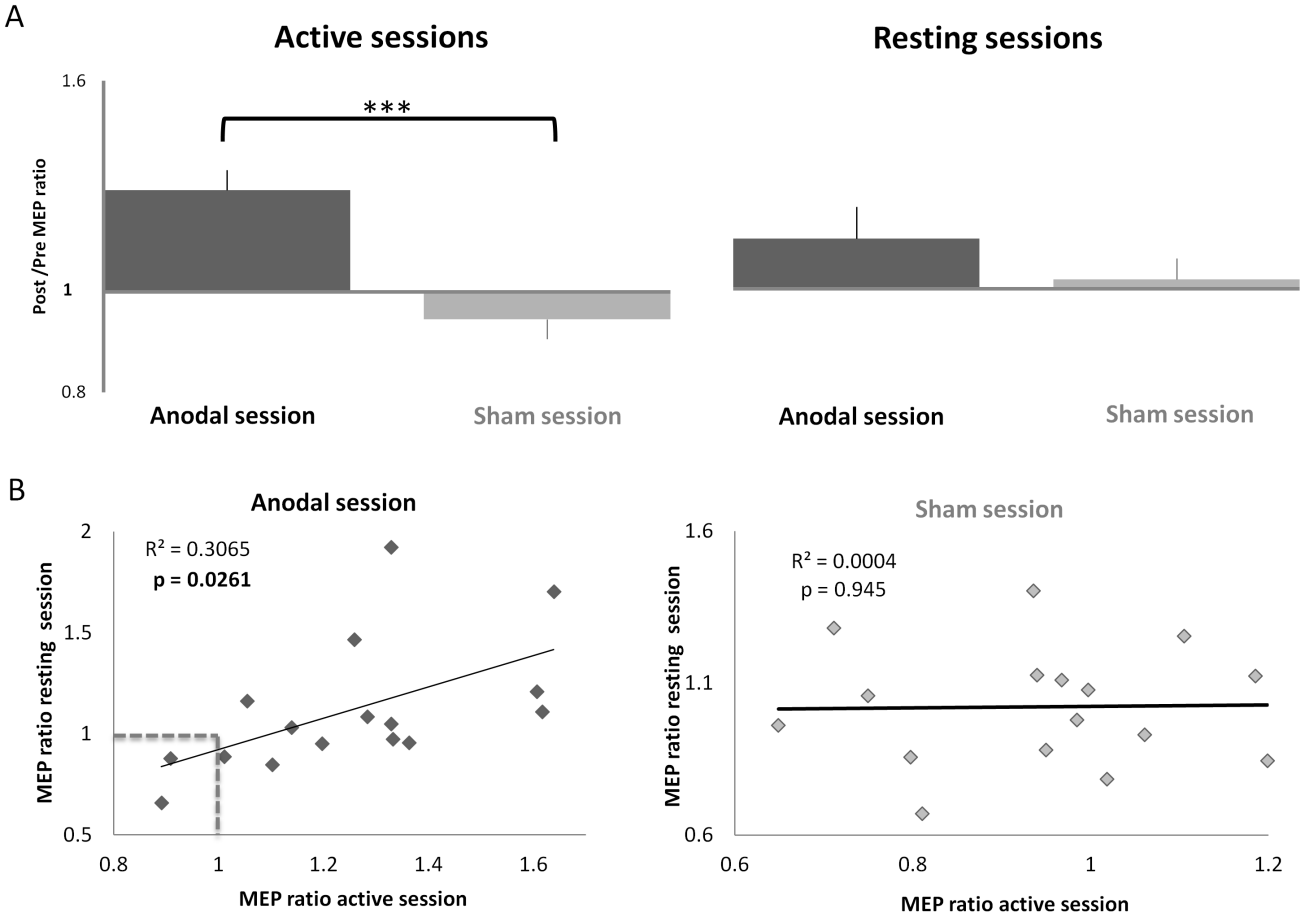
Corticomotor excitability results. **A**: MEP amplitude increased following the application of anodal tDCS in both active and resting motor states when compared to sham. The increase was only significant (p<0.001) in the active session. **B**: The MEP amplitude increase observed in the active and resting anodal sessions was significantly correlated (p = 0.026). This was not the case for the sham sessions. Data points to the left of and below the dotted lines (i.e. MEP ratio <1) indicate participants who did not increase excitability in either of the anodal sessions. Error bars represent mean ± SEM. *** p <0.001

In both anodal tDCS sessions, we observed increased MEP amplitudes following stimulation (relative to baseline) when compared to sham tDCS sessions (Fig. 3A). In the active anodal session, MEP amplitude increased 25±6% following tDCS application, which was significantly higher than in the sham session where a decrease of 6±4% was observed (paired *t*-test, anodal versus sham *t*_(15)_ = 4.47, p<0.001). There was also a significant increase from POST-TMS to PRE-TMS observed only in the active anodal session (paired *t*-test, PRE-TMS versus POST-TMS *t*_(15)_ = −3.74, p =  0.002). In the resting anodal session, we also observed an increase in MEP amplitude of 12±8% following tDCS application, but this effect did not reach significance when compared to the sham session where an increase of 2±5% was observed (paired *t*-test, anodal versus sham *t*_(15)_ = 1.08, p = 0.30). Furthermore, MEP variability (CV) was significantly reduced in the active sessions (0.30±0.02) compared to the resting sessions (0.49±0.01) (paired *t*-test, active versus resting *t*_ (15)_ = −6.179, p<0.001) but was not significantly modulated by tDCS (F _(1, 15)_ = 0.80, p = 0.39).

Across participants, the increase in cortciomotor excitability induced by tDCS was significantly correlated between the active and resting anodal session (r^2^ = 0.3065, p = 0.026), while no such relationship was observed for the sham sessions (r^2^  = 0.0004, p = 0.945) (Fig. 3B). It is also clear from Figure 3 that there is considerable individual variation in the response to anodal tDCS, with two participants exhibiting no or the opposite response expected based on previous literature (non-responders) in either the resting or active sessions (left and below the dotted lines).

### 2. Intracortical inhibition (SICI)

There was no significant difference between baseline intracortical inhibition in the anodal and sham sessions in either motor state (active motor state: anodal 67±7% vs sham 68±4%, paired *t*-test, anodal versus sham *t*_(15)_ = −0.12, p =  0.90; resting motor state: anodal 51±4% vs sham 53±5%, paired *t*-test, anodal versus sham *t*_ (15)_ = −0.69, p =  0.50).

**Figure 4:**
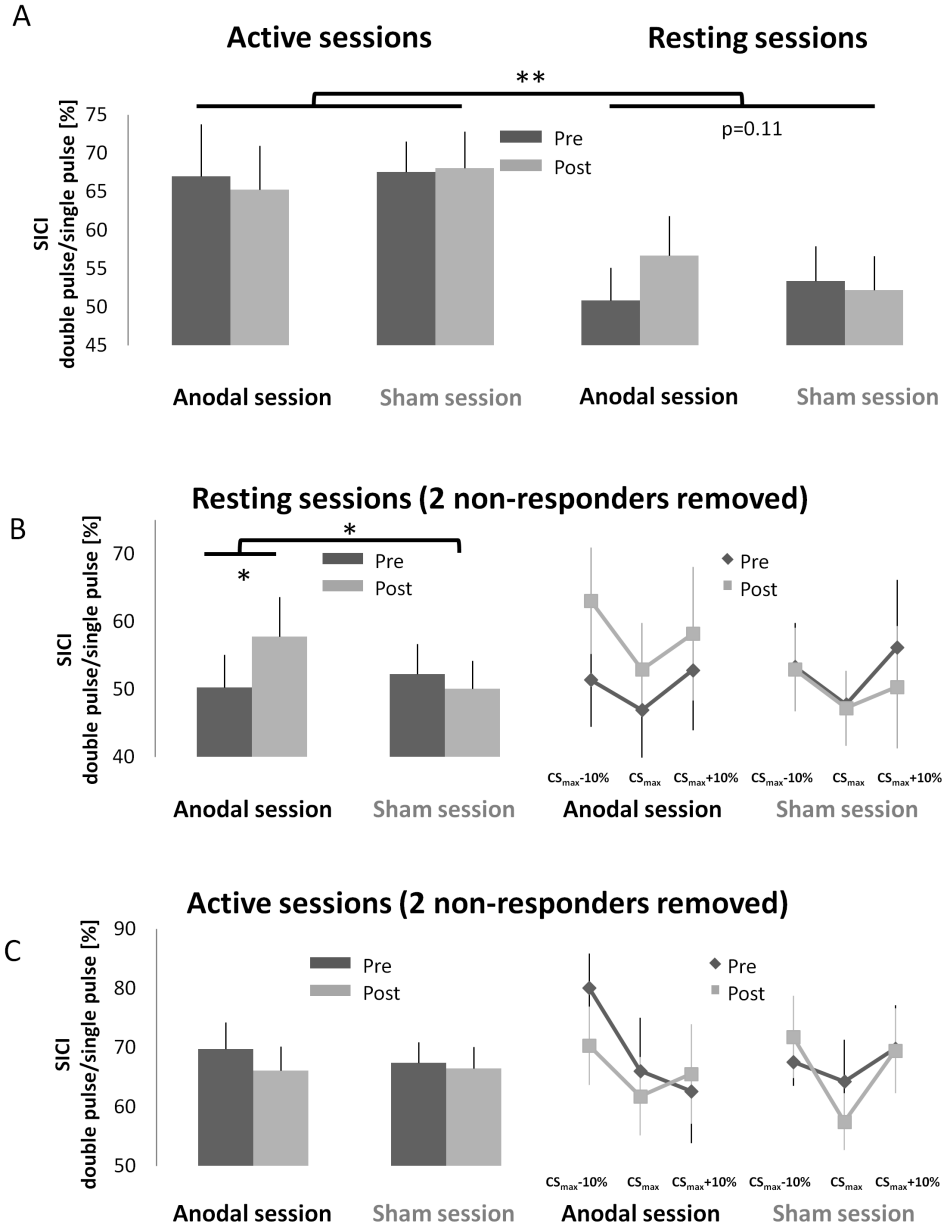
Intracortical inhibition (SICI) results. A: There was a significant decrease in SICI in the active motor state compared to the resting motor state (p = 0.004). There was a trend towards a reduction in SICI following anodal tDCS when non-responders were included in the analysis (p = 0.11). B: Mean SICI data and SICI curve from the resting sessions are shown when the 2 non-responders were removed from the analysis. The left panel shows the significant interaction between the anodal and sham sessions (p = 0.04). Post-hoc test revealed that SICI was significantly reduced in the anodal session (p = 0.027), but not the sham session (p = 0.478). The right panel shows the full SICI curves. C: Mean SICI data and SICI curve from the active sessions are shown when the 2 non-responders were removed from the analysis. There were no significant changes in either mean SICI data or SICI curve. Error bars represent mean ± SEM. * p<0.05; ** p <0.01.

In Figure 4A it can be observed that there was a reliable difference in the magnitude of SICI between motor states (paired *t*-test, active versus resting *t*_(15)_ = 3.40; p = 0.004), due to a reduction in intracortical inhibition in the active motor state. No other main effects or interactions were significant (F≤2.32, p≥0.12). SICI did not significantly change from baseline following either anodal (F _(1, 15)_ = 0.31, p = 0.58) or sham (F_(1, 15)_ = 0.04, p = 0.85) stimulation in the active motor state.

Next we focused on the effects of SICI in the resting state only, which were examined with a separate rm- ANOVA. Only in the anodal session was there a tendency for SICI to be lower at POST-TMS compared to PRE-TMS (PRE-TMS 51±4%; POST-TMS 57±5%). However, the rm-ANOVA revealed only a trend towards a TIME × STIMULATION interaction (F_(1, 15)_ = 2.85, p = 0.11; Fig. 4A right panel), while no other main effects or interactions approached significance (F≤1.8, p≥0.20). In an exploratory analysis performed for hypothesis generating purposes we excluded two participants that did not exhibit any response to anodal tDCS (see Fig. 3B, two data points separated by dashed lines). Note that this procedure uses a rather conservative criterion, i.e. the absence of any indication that tDCS increased corticomotor excitability. After excluding two non-responders a significant TIME × STIMULATION interaction (F_(1, 15)_ = 5.18, p = 0.04; Fig 4B left panel) was revealed suggesting that anodal tDCS reduced SICI (mean reduction of 8%±3% SEM) in those participants that exhibited an increase in corticomotor excitability in response to the stimulation (PRE-TMS 50±5%; POST-TMS 58±5%). Post hoc tests (Fisher's LSD) indicated a significant reduction in SICI at POST-TMS compared to PRE-TMS in the anodal session (p = 0.027; Fig. 4B left panel). There was no significant difference between different CS intensities (Fig. 4B right panel). Similar effects were not observed in the active session, neither with the full sample (Fig. 4A left panel) nor when the two non-responders were removed (Fig. 4C).

### 3. Background EMG

There was a significant difference in the bgEMG score between active and resting motor states (paired *t*-test, *t*_(15)_ = 8.94; p<0.001), however, no significant effects were observed for the factors STIMULATION and TIME (Table 2). This indicates that muscle activity was at a comparable level between conditions, a particularly important consideration for the active motor state in order to ensure that corticomotor excitability and SICI were measured under comparable conditions.

**Table 2:** Background EMG (mV).

	Rest-Anodal	Rest-Sham	Active-Anodal	Active-Sham
**PRE-TMS**	0.0017±0.0007	0.0020±0.0009	0.0687±0.0334	0.0702±0.0304
**POST-TMS**	0.0018±0.0009	0.0019±0.0010	0.0673±0.0308	0.0709±0.0328

Values are means ± SEM.

###  4. Cortical silent period and wrist extension force

Table 3 summarizes cSP and wrist extension force data in the active sessions. The cSP was not significantly altered by tDCS (anodal vs sham session: F_(1, 15)_ = 0.169, p = 0.687), nor did it differ between PRE- and POST-TMS measurements (anodal session: F_(1, 15)_ = 0.03, p = 0.87; sham session: F_(1, 15)_ = 0.23, p = 0.64). Statistical tests confirmed that there were also no differences in wrist extension force between anodal and sham tDCS sessions (F_ (1, 15)_ = 1.392, p = 0.256) or between PRE- and POST-TMS measurements (anodal session: F_ (1, 15)_ = 0.093, p = 0.765; sham session: F_(1, 15)_ = 0.454, p = 0.511).

**Table 3:** Cortical silent period and wrist extension force.

		cPS (ms)	Max Force (N)	Force during TMS (N)
**ANODAL**	PRE-TMS	55±5	207.16±13.65	18±1
	POST-TMS	55±6		18±1
**SHAM**	PRE-TMS	53±5	202.24±12.07	17±1
	POST-TMS	54±6		17±1

Values are means ± SEM.

### 5. Questionnaire

The number of hours sleep in the night prior to testing was not significantly different across sessions (F_(3, 45)_  = 0.265, p = 0.850; table 4). The discomfort score did not differ reliably between anodal and sham sessions (F_(1, 15)_ = 2.54; p = 0.13; table 4), suggesting that participants did not perceive the difference between stimulation conditions.

**Table 4:** tDCS discomfort score and sleeping hours.

	Rest-Anodal	Rest-Sham	Active-Anodal	Active-Sham
**tDCS discomfort (0-10)**	1.53±0.25	1.16±0.25	1.59±0.26	1.44±0.25
**Sleeping hours (hrs)**	7.44±0.36	7.75±0.37	7.42±0.34	7.41±0.46

Values are means ± SEM.

## Discussion

It is well established that anodal tDCS applied to the hand area of primary motor cortex can significantly increase corticomotor excitability when measured at rest [1], [9], [11], [14], [15], [18], [21], [24]. Here we extend on previous work by demonstrating that similar anodal tDCS aftereffects are present when targeting a more proximal upper limb muscle (ECR) during an active motor state. In general this result is consistent with the relatively few previous studies that have measured the aftereffects of anodal tDCS on corticomotor excitability in an active target muscle. Hendy and Kidgell [16] recently reported increased corticomotor excitability in pre-contracted ECR following three weeks of strength training combined with 2 mA anodal tDCS. Jeffery et al [35] observed increased corticomotor excitability in pre-contracted muscles of the lower leg following 10 min of 2 mA anodal tDCS.

In the present study we only observed a trend towards increased corticomotor excitability in the resting motor state following anodal tDCS, which was not significant when compared to sham. Our results are consistent with Uy and Ridding [24] who tested the aftereffects of anodal tDCS in the first dorsal interosseous, abductor digiti minimi and flexor carpi ulnaris in healthy adults. They observed a significant difference in response to anodal tDCS in the first dorsal interosseous, but not in the forearm muscle (i.e. flexor carpi ulnaris). Another study applying 1 mA cathodal tDCS to the infraspinatus region in the left primary motor cortex showed that cathodal tDCS decreased corticomotor excitability of distal hand muscles but not the proximal shoulder muscles [36]. This is in line with a previous study that applied theta burst stimulation to different regions of the motor cortex and found that the results obtained from the biceps muscle are highly variable compared to the same stimulation applied to a hand muscle (FDI). The authors pointed out that the magnitude and reliability of theta burst stimulation depends on which cortical region is targeted [37]. Together, previous work raises the possibility that the corticomotor excitability of more proximal muscles might be less sensitive to modulation through brain stimulation, possibly because intrinsic hand muscles have larger representations in human primary motor cortex[38], [39] and are controlled more selectively than forearm muscles [40]. Moreover, our data indicate that much depends on how the aftereffects of tDCS are assessed. One aspect of general consideration when using TMS is that evoking MEPs while the subject is at rest provides good control of muscular activity but poor control about the psychophysiological state of the subject. It has been shown previously that MEP amplitudes are influenced by arousal, visual attention[41], sleepiness [42], vegetative state [43] and seemingly irrelevant visual background information [44]. Even though we tried to control these aspects during each session in addition to including a sham condition in the design, TMS measurements at rest might still be subject to greater noise due to non-specific effects. This would lead to reduced sensitivity with small modulations of corticomotor excitability failing to reach significance. This argument is supported by two of our findings: First, it is worth noting that we observed a significant difference in MEP size variability between the active and resting motor states, with the CV being significantly lower in the active motor state. Therefore it is also possible that in our data higher variability in corticomotor excitability measurements at rest prevented statistical significance being reached. Second, we also observed that the change in corticomotor excitability in active and resting anodal tDCS sessions was positively correlated. That is, interindividual differences in the response to tDCS were largely maintained across assessments in the active and the resting motor state.

Of interest is the fact that not all participants responded to anodal tDCS. We tested 16 participants and the corticomotor excitability of 2 (12.5%) did not respond to stimulation in either anodal session. These participants did not differ from the rest of the sample in terms of baseline measurements, such as sleep hours, physical activity prior to the testing sessions and self reported tDCS discomfort scores. This observation is in line with previous reports of inter-individual variability in response to anodal tDCS: Nitsche and Paulus [1] tested 12 participants and the increase in corticomotor excitability ranged from 0 to approximately 200%, with an average increase of about 50%. Another study which quantified the effects of anodal tDCS with recruitment curves reported that slopes increased for 4 out of 5 participants [6]. When cortical current flow was modeled using the same tDCS parameters as in our study, differences were found between participants [45]. These studies (and ours) used 1 mA current applied to the primary motor cortex, which might not be sufficient to consistently induce sufficient current flow in the brain, particularly when there are substantial variations in anatomy between participants [45], [46]. This suggests that future studies should consider the use of subject specific intensities in order to induce more robust effects [45]. It is also possible that a substantial proportion of the population does not respond to tDCS. Our finding identifying 12.5% non-responders is in line with two recent studies showing that there is considerable inter-individual variability in response to tDCS. Wiethoff et al [47] reported that approximately 25% of participants did not show an increase in corticomotor excitability following the application of 10 min of 2 mA anodal tDCS [47] and Lopez-Alonso et al [48] found that about 50% of participants showed no increase in corticomotor excitability after the application of 13 min of 1 mA anodal tDCS [48]. Our criteria for determining non-responders is similar to the above studies but slightly more conservative, namely we only classified participants as non-responders if they demonstrated no increase in both the resting and active anodal sessions. Together, recent studies suggest that a substantial proportion of the population does not respond to anodal tDCS with an increase in corticomotor excitability. Depending on the criteria and which stimulation/test protocol is applied, the proportion of non-responders appears to vary between 12.5 and 55%.

We observed an approximate 15% decrease in SICI in the active motor state compared to the resting motor state. This was expected based on previous work showing that the strength of intracortical inhibition is reduced when measured with a voluntary tonic contraction of the target muscle [31], [49]. Anodal tDCS did not lead to any changes in SICI when tested during the active motor state, most likely due to the large reduction in inhibition that might have masked more subtle modulations of SICI caused by tDCS. SICI was decreased following anodal tDCS when tested in the resting motor state, but this decrease did not differ significantly from the sham condition, unless the two participants that did not respond to anodal tDCS in any of the two sessions were excluded from the analysis. We did not match MEP size evoked by the TS across PRE and POST sessions. TS amplitude influences the level of SICI [50], [51] and some studies have demonstrated that matching TS is important for accurately measuring changes in SICI post-intervention [52]. However, other experiments have indicated a lesser effect of matching TS amplitude [53], [54]. Moreover, we observed a trend towards reduced SICI only when tested in a resting motor state, i.e. when TS MEP amplitude changed only to an insignificant extent from PRE to POST. Previous studies measuring the influence of tDCS on SICI in hand muscles have revealed divergent results: Nitsche et al [11] found a significant decrease in intracortical inhibition following 13 min of 1 mA anodal tDCS. Another study applied 20 min of 2mA anodal tDCS to the ECR region in the primary motor cortex and showed a significant decrease in SICI when participants contracted their muscle with 20% and 50% of MVC, but no decrease during a lighter muscle contraction (5% MVC) [16]. Hummel et al [6] reported reduced intracortical inhibition after applying 20 min of 1 mA anodal tDCS to chronic stroke patients. Conversely, Batsikadze et al [15] recently observed no change in SICI following 20 min of 2 mA anodal tDCS. Notably, previous comparisons of SICI between upper limb muscles found that the amount of inhibition in proximal muscles to be significantly less than that observed in the intrinsic hand muscles [55], [56]. Furthermore, when SICI is measured at rest it is known to suffer from substantial inter-individual variability. In an attempt to account for this we measured SICI intensity curves, which are thought to provide a better indication of intracortical inhibition compared to a single measurement. We observed a decrease of about 8% in SICI, which is in line with previous studies that have reported SICI decreases of between 7∼15% [9], [11], [16] following anodal tDCS. When considered in combination with previous work, our findings suggest that tDCS has a less robust influence on SICI in comparison to corticomotor excitability, even when SICI curves are measured. This might result from larger inter-individual variability, lower responsiveness of ECR to the SICI protocol and/or methodological details that might have influenced SICI results. More specifically, most studies cited above as well as ours used an interstimulus interval (ISI) of 3ms between the CS and TS. However, this ISI might have been suboptimal because there is a risk that SICI measurements were confounded by short interval cortical facilitation [57]. Future studies should use a shorter ISI and larger cohorts to determine whether anodal tDCS affects SICI to a relevant extent and which proportion of the population responds to tDCS with changes in SICI.

There was no significant difference in cSP between anodal and sham sessions. SICI and cSP are both measures of intracortical inhibition and are thought to represent inhibitory processes mediated by different (but not mutually exclusive) pathways. SICI measures activation of the intracortical inhibitory pathways that reduce the excitability of corticospinal neurons within primary motor cortex [23], [50], [58], mainly depends on GABA_A_ receptors [23] and seems to be responsive to anodal tDCS[5]. By contrast, cSP mainly depends on GABA_B_ receptors [59] and does not seem to be modulated by anodal tDCS [15], [16], [60], a finding that is consistent with our results.

Finally, we note that there are some limitations to our study. First, we did not measure IO-curves to estimate the aftereffect of anodal tDCS and we did not test long-term aftereffects (>10min). However, since subjects had to produce a controlled force we decided to keep the protocol per session short to prevent muscular and mental fatigue. Second, only one forearm muscle was tested in this study, thus we could not directly compare the response of proximal versus distal muscles. Finally, our results concerning the influence of tDCS on SICI are unconvincing because we observed only a small effect when SICI was measured in the resting ECR, which is at odds with the tDCS induced increase of corticomotor excitability that was only measured when the muscle is activated. Future experiments investigating the influence of tDCS on SICI might benefit from a modified experimental protocol using a shorter ISI of 2 ms for the paired-pulse measurements and matching the MEP amplitude evoked by the TS across PRE and POST measurements.

Here we aimed to show the feasibility of measuring changes in corticomotor excitability and intracortical inhibition in the forearm area of primary motor cortex. While most previous tDCS studies targeted the intrinsic hand muscles, we investigated a proximal upper limb muscle (ECR) in order to optimize clinical relevance. We found that anodal tDCS applied to the primary motor cortex at rest induced similar aftereffects in ECR as those previously reported for the intrinsic hand muscles, however, only when aftereffects were measured while subjects performed a controlled muscle activation. This confirms that targeting more proximal muscles of the upper limb with tDCS is achievable. Furthermore, we demonstrated that the effects of anodal tDCS on corticomotor excitability can be appropriately assessed during a low-level controlled contraction of the target muscle. This outcome is positive for future studies in clinical populations such as stroke survivors, where TMS induced MEPs are mostly absent during rest.
